# Nodules that Wax and Wane: Unusual Presentation of Amiodarone Lung Toxicity

**DOI:** 10.7759/cureus.9058

**Published:** 2020-07-08

**Authors:** Candace Griffith, Samia Hossain, Arun Minupuri, Michael Korman

**Affiliations:** 1 Internal Medicine, Mercy Hospital, Yeadon, USA; 2 Internal Medicine, Mercy Catholic Medical Center, Darby, USA; 3 Pulmonology, Mercy Catholic Medical Center, Darby, USA

**Keywords:** amiodarone, pulmonary toxicity, waxing and waning of nodules

## Abstract

Amiodarone is associated with a wide variety of side effects, but unusual presentations can make it difficult to diagnose and treat. This case report describes amiodarone causing nodular changes in the lung, as opposed to diffuse interstitial disease. After cessation of the medication, there was marked radiographic improvement in the nodules.

## Introduction

Amiodarone is a commonly prescribed drug that is effective in treating cardiac arrhythmias, mainly ventricular and supraventricular arrhythmias [1]. Caution needs to be exercised as the drug also has a high incidence of side effects, ranging from photosensitivity to toxicities of the liver, thyroid, and lung [2]. Amiodarone induced pulmonary toxicity (AIPT) has an incidence of about 5%-10%, and it is considered the most serious adverse effect with mortalities reaching up to 10% of the patients [3-4]. These changes in the lungs are commonly seen with chronic use of the drug for months to years and with dosages of ≥400 mg/day [5]. We describe a case report of an individual who was on 200 mg/day of amiodarone for approximately five years.

## Case presentation

We present a case of a 79-year-old male with a past medical history of cardiac arrest in 2009 (with subsequent placement of automatic implantable cardioverter-defibrillator (AICD)), diastolic heart failure, and obstructive sleep apnea on continuous positive airway pressure (CPAP) therapy who first presented to us in 2014 having just been treated for possible pneumonia and diagnosed with asthma. He was a nonsmoker and had no occupational exposures. Chest examination at that time showed basilar crackles although CT chest did not show any evidence of diffuse interstitial disease. It did show however two irregular ground-glass type nodular densities each of 12 mm in the right upper and right lower lobe. The patient's medication at that time included an inhaled corticosteroid, amiodarone, amlodipine, aspirin, atorvastatin, furosemide, and metoprolol. The patient had extensive serum testing looking for causes of interstitial or inflammatory lung disease all with negative results.

**Figure 1:**
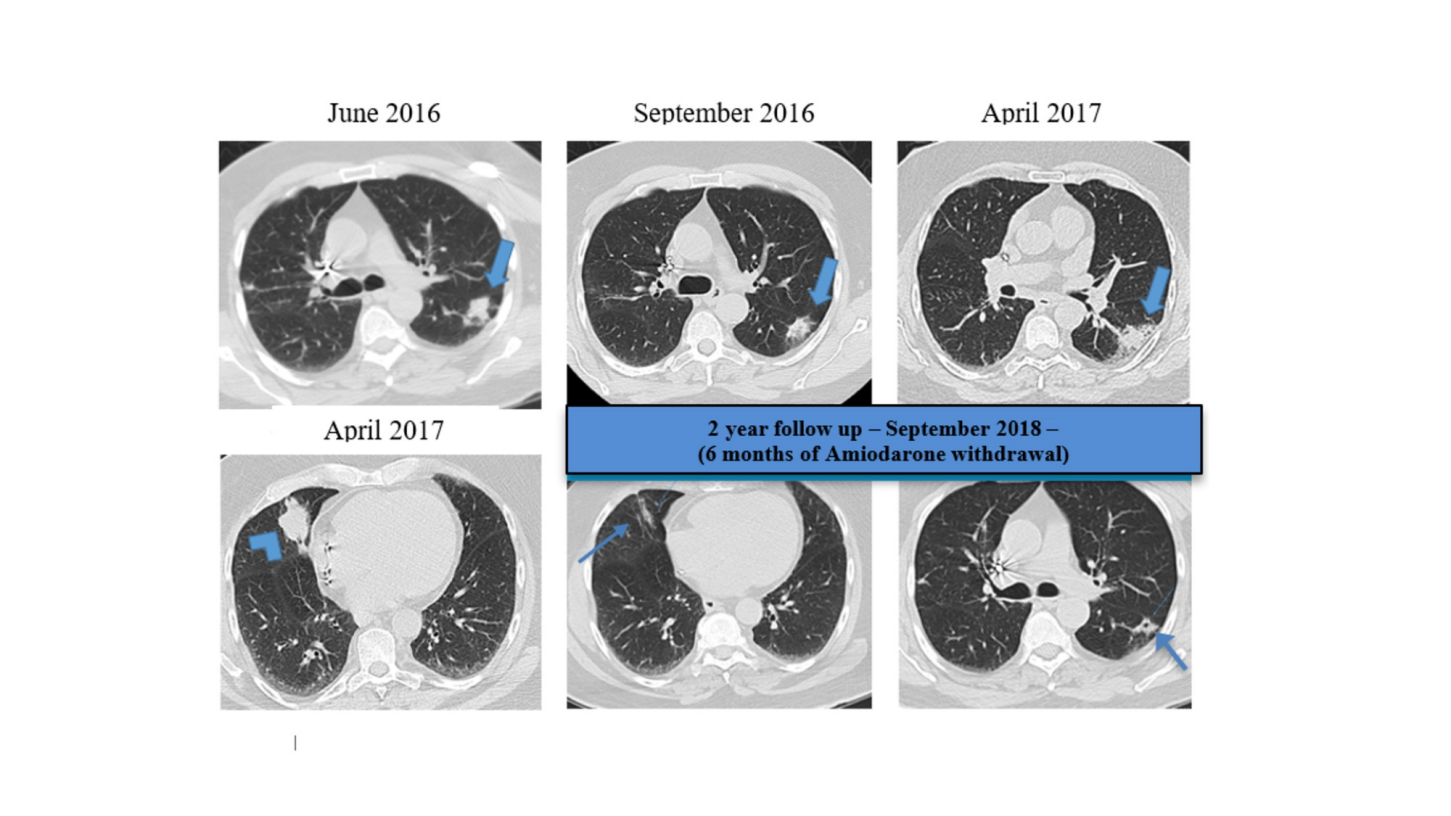
CT scans demonstrating waxing and waning of nodules CT scans demonstrating waxing and waning consolidations, predominantly affecting the superior aspect of the left lower lobe, with decreased conspicuity in September 2016 and increased size in April 2017 (thick arrows) when there was also the development of a new consolidation at the right middle lobe (arrowhead). In September 2018 (six months after amiodarone withdrawal), there was a resolution of right middle lobe consolidation with only linear areas of scar remaining and a significant decrease of the left lower lobe consolidation (thin arrows).

Over the next several years, the patient had a number of waxing and waning nodular infiltrates with one measuring 3.9 cm in its largest diameter within the right middle lobe and another pleural-based density in the left lower lobe approximately 2 cm in size (Figure 1). The right upper and right lower nodular densities also persisted with varying sizes. The patient never exhibited typical asthma symptoms nor was he found to have significant bronchospasm, although he did have some intermittent nonspecific cough and sputum production. He was able to be tapered off his inhaled corticosteroid.

The patient underwent bronchoscopy with associated washings, brushings, and transbronchial lung biopsies. They were all negative as were cultures for bacteria, fungus, and tuberculosis. 

In March of 2018, amiodarone was discontinued. By September of 2018, there was marked radiographic improvement.

## Discussion

Amiodarone has a long half-life and a high tissue affinity for the lung; it accumulates in type 2 pneumocytes [6]. Mono-n-desethylamiodorone, an active metabolite of amiodarone, exhibits cytotoxic activity and accumulates in the lungs even more so than amiodarone [7]. It has been suggested that both direct toxic injury to lung cells and also an indirect immunologic reaction is responsible for lung injury [6].

The mechanisms involved in AIPT are not well understood. The hypothesis of a direct toxic reaction is supported by amiodarone pulmonary and neurotoxicity being usually dose related, the accumulation of cellular drug‐phospholipid complexes that interferes with normal cellular metabolic pathways disrupting cellular and organelle membrane function, as well as generation of toxic oxygen species by amiodarone [8].

This case showed an unusual presentation for amiodarone pulmonary toxicity with waxing and waning nodular infiltrates. It is important to note that his type of presentation can mimic that of other inflammatory pulmonary processes or malignancy. Cessation of amiodarone was associated with resolution of pulmonary nodules and this is considered to be the mainstay of treatment for uncomplicated AIPT.

## Conclusions

Clinicians need to consider medication induced as part of their differential for patients on amiodarone having respiratory symptoms with unusual radiographic findings. Once an accurate diagnosis is attained, appropriate treatment in the form of cessation of the drug can lead to a favorable response from patients.
